# Moderate COVID-19: Clinical Trajectories and Predictors of Progression and Outcomes

**DOI:** 10.3390/jpm12091472

**Published:** 2022-09-08

**Authors:** Apostolos G. Pappas, Andreas Panagopoulos, Artemis Rodopoulou, Michaella Alexandrou, Anna-Louiza Chaliasou, Konstantinos Skianis, Eleftheria Kranidioti, Eleftheria Chaini, Ilias Papanikolaou, Ioannis Kalomenidis

**Affiliations:** 1 1st Department of Critical Care & Pulmonary Service, School of Medicine, Evangelismos General Hospital, National and Kapodistrian University of Athens, 10676 Athens, Greece; 2 COVID-19 Unit, “Evangelismos” General Hospital, 10676 Athens, Greece; 3 3rd Department of Internal Medicine, “Evangelismos” General Hospital, 10676 Athens, Greece; 4 4th Department of Internal Medicine, “Evangelismos” General Hospital, 10676 Athens, Greece; 5 Data Science & Mining Team, École Polytechnique, 91120 Paris, France; 6 Pulmonary Department, Corfu General Hospital, 49100 Corfu, Greece; 7 COVID-19 Unit, Corfu General Hospital, 49100 Corfu, Greece

**Keywords:** COVID-19, SARS-CoV-2, moderate disease, risk factors

## Abstract

Background: Patients with COVID-19 commonly present at healthcare facilities with moderate disease, i.e., pneumonia without a need for oxygen therapy. Aim: To identify clinical/laboratory characteristics of patients with moderate COVID-19, which could predict disease progression. Methods: 384 adult patients presented with moderate COVID-19 and admitted to two hospitals were retrospectively evaluated. In a multivariate analysis gender, age, BMI, Charlson Comorbidity Index (CCI) and National Early Weaning Score 2 were treated as co-variates. The development of hypoxemic respiratory failure, intubation rate and risk of death were considered as dependent variables. Estimated values are presented as odds-ratio (OR) with 95% confidence interval (CI). Results: Most of the patients were male (63.28%) with a mean (standard deviation) age of 59 (16.04) years. Median (interquartile range) CCI was 2 (1–4). A total of 58.85% of the patients developed respiratory failure; 6.51% were intubated, and 8.85% died. The extent of pneumonia in chest X-ray (involvement of all four quartiles) [OR 3.96 (1.18–13.27), *p* = 0.026], respiratory rate [OR 1.17 (1.05–1.3), *p* = 0.004], SatO_2_ [OR 0.72 (0.58–0.88), *p* = 0.002], systolic blood pressure [OR 1.02 (1–1.04), *p* = 0.041] and lymphocyte count [OR 0.9993 (0.9986–0.9999), *p* = 0.026] at presentation were associated with the development of respiratory failure. The extent of pneumonia [OR 26.49 (1.81–387.18), *p* = 0.017] was associated with intubation risk. Age [OR 1.14 (1.03–1.26), *p* = 0.014] and the extent of pneumonia [OR 22.47 (1.59–316.97), *p* = 0.021] were associated with increased risk of death. Conclusion: Older age, the extent of pneumonia, tachypnea, lower SatO_2_, higher systolic blood pressure and lymphopenia are associated with dismal outcomes in patients presenting with moderate COVID-19.

## 1. Key Messages

*What is the key question?* What are the clinical/laboratory characteristics and the predictors of disease progression in patients with moderate COVID-19 (i.e., patients with COVID-19 pneumonia and without respiratory failure)?

*Which are the main findings?* Older age, tachypnea, lower SatO_2_, higher systolic blood pressure, lymphopenia and the extent of pneumonia in a chest X-ray at presentation were independently associated with dismal outcomes in patients with moderate COVID-19.

*Why read on?* The present study focuses on patients with moderate COVID-19 at presentation and may help to identify those patients at risk for disease progression and hospitalization.

## 2. Introduction

Nearly two years after COVID-19 first appeared in Wuhan, China, [1] SARS-CoV-2 infection still poses a major threat worldwide. Since multiple new variants have emerged and vaccination programs have not met their goals [2], morbidity and mortality due to COVID-19 are expected to remain high [3]. Many regions will probably face a resurgence of COVID-19 [4], which will cause overwhelming pressure to hospital systems [3]. In an effort to make optimal use of hospital resources, it is crucial to be able to predict disease progression [3,5]. This is particularly important for patients presenting with moderate disease (patients with clinical and/or radiographical evidence of pneumonia with no need for supplemental oxygen, i.e., SpO_2_ ≥90% on room air) [6], since decisions on their admission to the hospital are intriguing. Unfortunately, the currently available prognostic tools are suboptimal in predicting clinical deterioration, especially in the medium-risk population [7,8].

In the present study, we analyzed data collected from patients presenting to the emergency department (ED) with moderate COVID-19 [6] and admitted to the hospital. Our aim was to describe their clinical course and identify factors associated with disease progression and outcomes. We hypothesized that certain clinical and/or laboratory features available at the time of first evaluation of these patients exhibit important predictive values.

## 3. Patients and Methods

We retrospectively evaluated 384 consecutive patients with age > 18 and confirmed SARS-CoV-2 infection by quantitative RT-PCR in nasopharyngeal swab specimens, who presented with moderate COVID-19 [6] and were admitted to the hospital. Among them, 41 patients were admitted to the COVID-19 ward of “Corfu General Hospital”, Corfu, Greece, between 1 September 2020 and 30 April 2021, while 343 were admitted to the COVID-19 ward of “Evangelismos” General Hospital, Athens, Greece, between 1 September 2020 and 31 December 2020. Patients’ demographical and clinical characteristics, laboratory values and final outcomes were retrieved from medical records. Quantitative evaluation of chest X-ray pathological lesions was performed as described elsewhere [9]. Due to the retrospective nature of the study, an informed consent was not obtained by the patients. The study was approved by the Ethics Committees of the Hospitals. Protocol numbers: 44 (25-2-2021) and 1615B (13-4-2021).

### Statistical Analysis

Quantitative variables with Gaussian distribution are presented as mean (standard deviation-SD) and were analyzed with Student’s T-test or one-way analysis of variance (ANOVA). Non-parametric quantitative variables are presented as median (interquartile range-IQR) and were analyzed using the Mann–Whitney U-Test. Qualitative variables are presented as n (%) and were analyzed with a Chi-square test. All variables were evaluated using two-tailed tests. A univariate analysis was performed with the development of hypoxemic respiratory failure, intubation and death being the dependent variables. Subsequently, the statistically significant variables in univariate analysis were evaluated in a multiple logistic regression model with gender, age, body mass index (BMI), Charlson comorbidity index (CCI) [10] and national early weaning score (NEWS) 2 [8] treated as covariates, while the development of respiratory failure, intubation and death were considered as dependent variables. Calculated estimates are shown as odds ratios (OR), with 95% confidence intervals (CI). For quantitative variables, OR reflects changes per given unit. The statistical analysis and regression tasks were performed with Python and open-source libraries: scikit-learn and stats models.

## 4. Results

### 4.1. Patients’ Clinical and Laboratory Characteristics

A total of 384 patients with moderate COVID-19 at presentation were evaluated, 243 of whom (63.28%) were male. Most of them were of European origin (83.07%) with a mean (SD) age of 59 (16.04) years. The median (IQR) CCI score was 2 (1–4). The median (IQR) number of days from symptom onset to presentation was 6 (4–8). The median (IQR) NEWS2 score at presentation was 2 (1–5). Comorbidities, symptoms/signs and laboratory values are depicted in Table 1, Table 2 and Table 3.

### 4.2. Treatments, Clinical Trajectories and Outcomes

The treatment of SARS-CoV-2 infection during patients’ hospital stay was given in accordance to the national guidelines at the certain period of time (eody.gov.gr/en/covid-19/ accessed on 27 August 2020). Briefly, patients progressing to hypoxemic respiratory failure received therapy with intravenous (IV) dexamethazone and IV remdesivir (unless contraindicated). Most of them received antibiotic treatment (91.67%), with azithromycin being the most common antibiotic (72.39%). The majority of the patients received anticoagulants in a prophylactic dose (86.95%), mostly with subcutaneous (sc) low-molecular weight heparine (72.32%) and sc fondaparinux (20.63%). Some 3.92% received no anticoagulants due to hemorrhage or low platelet counts, and 8.88% received therapeutic anticoagulation due to chronic conditions (e.g., atrial fibrillation) or newly diagnosed venous thromboembolism. The median (IQR) number of hospitalization days was 10 (7–14.25). Major complications are described in Table 4.

Within 2 (1–4) days of hospital admission, 226 patients (58.85%) deteriorated and developed hypoxemic respiratory failure, requiring supplemental oxygen. A total of 49 (12.76%) of patients required therapy with high-flow nasal oxygen (HFNO), and 27 (7.03%) were treated with non-rebreathing masks (NRM). Subsequently, 41 patients (10.68%) were admitted to the intensive care unit (ICU), and 25 (6.51%) patients required endotracheal intubation and mechanical ventilation. A total of 21 of them eventually died of respiratory failure or ICU-related complications. Another 13 patients with Do-Not-Intubate (DNI) orders died from respiratory failure. Overall, 34 patients (8.85%) died.

### 4.3. Link between Clinical/Laboratory Features and Disease Progression or Outcomes

**Figure 1:**
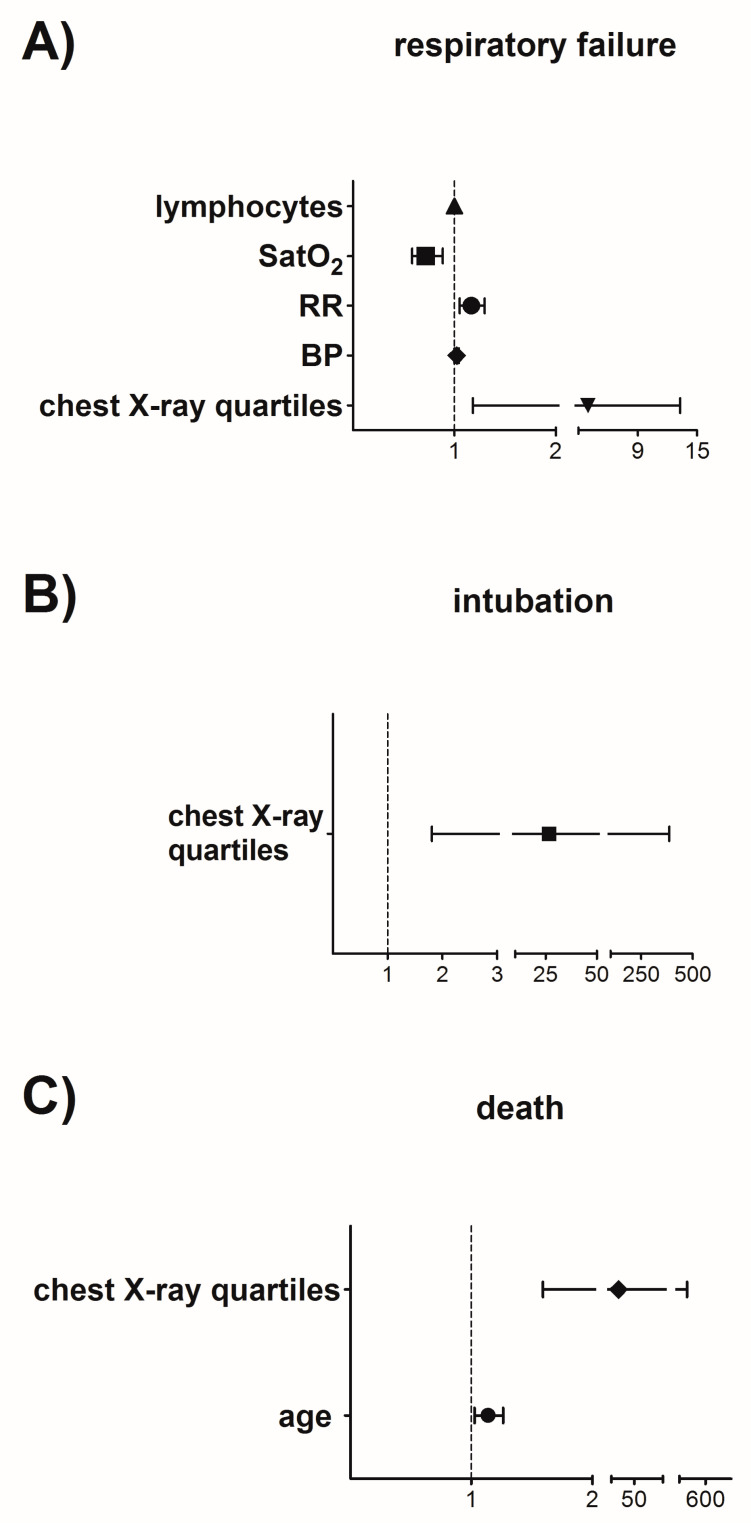
**Multivariate analysis-Forest Plots.** Statistically significant variables in univariate analysis were evaluated in a multiple logistic regression model with gender, age, body mass index (BMI), Charlson comorbidity index (CCI), and national early weaning score (NEWS) 2 treated as covariates, while progression to respiratory failure, intubation and death were considered as dependent variables: (**A**) involvement of all four chest X-ray quartiles (*p* = 0.026), higher systolic blood pressure [mmHg, (*p* = 0.041)], higher respiratory rate [breaths per min, (*p* = 0.004)], lower SatO_2_ [%, (*p* = 0.002)] and lymphopenia [cells/μL, (*p* = 0.026)] were independent risk factors of the development of respiratory failure; v (**B**) involvement of all four chest X-ray quartiles (*p* = 0.017) was an independent predictor of intubation; (**C**) older age [years, (*p* = 0.014)] and involvement of all four chest X-ray quartiles (*p* = 0.021) were independent predictors of death. Calculated estimates are shown as odds ratios (OR), with 95% confidence intervals (CI). For quantitative variables, OR reflects changes per given unit. *p*-values < 0.05 were consider significant.

The association between clinical/laboratory features at presentation and the development of respiratory failure, intubation and death were assessed using univariate and multivariate analysis. Independent variables significantly associated with at least one dependent variable in the univariate analysis are presented in Supplementary Tables S1–S3. Significant values were further evaluated in a multiple regression model, with gender, age, BMI, CCI and NEWS2 serving as co-variates. The involvement of all four chest X-ray quartiles [OR 3.96 (1.18–13.27), *p* = 0.026], systolic blood pressure [OR 1.02 (1–1.04), *p* = 0.041], respiratory rate [OR 1.17 (1.05–1.3), *p* = 0.004], SatO_2_ [OR 0.72 (0.58–0.88), *p* = 0.002] and blood lymphocyte count [OR 0.9993 (0.9986–0.9999), *p* = 0.026] at presentation were independent predictors of the development of hypoxemic respiratory failure (Figure 1A). The involvement of all four chest X-ray quartiles [OR 26.49 (1.81–387.18), *p* = 0.017] was also independently associated with intubation risk (Figure 1B). Finally, age [OR 1.14 (1.03–1.26), *p* = 0.014] and involvement of all four chest X-ray quartiles [OR 22.47 (1.59–316.97), *p* = 0.021] were independent predictors of death (Figure 1C). Of note, only the size of pneumonia on chest imaging was consistently associated with all three different outcomes in the multiple regression analysis.

## 5. Discussion

In this retrospective analysis, we evaluated 384 patients admitted with moderate COVID-19 [6] to two different hospitals in Greece. Most of the patients were of European origin, with a mean age of 59 years and a normal BMI. Arterial hypertension was the most frequent comorbidity, and the median (IQR) CCI was 2 (1–4). Over half of the patients developed hypoxemic respiratory failure, at a median (IQR) 2 (1–4) days after admission, with 10.68% requiring ICU admission and 6.51% requiring invasive mechanical ventilation; 8.85% of patients died. Multiple regression analysis revealed that: (1) involvement of all four chest X-ray quartiles, high systolic blood pressure and respiratory rate, low SatO_2_ and low number of blood lymphocytes at presentation were significantly associated with high risk of development of respiratory failure; (2) involvement of all four chest X-ray quartiles was significantly associated with increased risk of intubation; and (3) older age and the involvement of all four chest X-ray quartiles were significantly associated with increased risk of death.

To our knowledge, this is the first study focusing entirely on patients with moderate COVID-19 at presentation. Although these patients were not in urgent need for hospital admission, they were admitted to both hospitals during the study period when presenting with significant hypoxemia (SaO_2_ < 94%) or multiple/severe co-morbidities. This approach was mainly based on the difficulty to estimate the individual patient risk, especially if one considers that SARS-CoV-2 infection is characterized by a highly variable clinical course, and worsening of respiratory function may appear several days after symptom onset [11]. While the high rate of rapid (usually within 48 h after admission) progression to respiratory failure (58.85%) observed in our cohort partly justifies this practice, still more than 40% of patients could be treated as outpatients, in this way reducing patient discomfort and pressure on the hospital system during epidemic surges [2,4].

In an effort to predict clinical deterioration, several prognostic tools have been previously developed for COVID-19 [7,8], but they appear suboptimal when applied to medium-risk populations [7]. Similarly, we observed that CCI and NEWS2 scores were not associated with any of the outcomes tested in the multiple regression analysis. A common characteristic of these clinical severity scores is that they do not take into account the size of pneumonia on chest imaging, a feature that in our study was consistently associated with the development of respiratory failure, the risk of intubation and death. The close link between lung imaging findings and the disease progression and outcomes is in agreement with previous reports in COVID-19 patients (with varying disease severity), demonstrating that outcomes could be predicted by baseline chest radiography [9,12] and bedside lung ultra-sonography [13]. Thus, patients with extensive bilateral pneumonia at presentation should most likely be admitted to the hospital, even if they have not yet developed respiratory failure.

Apart from a chest X-ray, other respiratory parameters independently associated with the development of hypoxemic respiratory failure were lower SatO_2_ and higher respiratory rate at presentation. In contrast, dyspnea was not associated with disease progression, more likely reflecting the fact that several COVID-19 patients do not report this symptom in spite of the severity of the respiratory disease [14]. The association of higher systolic blood pressure with the development of respiratory failure comes in line with previous reports describing that patients on anti-hypertensive medication exhibited lower risk of in-hospital mortality [15]. Similarly, the severity of lymphopenia [16] and older age [17] have been previously well-characterized predictors of outcomes in unselected patients with SARS-CoV-2 infection.

Apart from its retrospective design, an important limitation of our study is the fact that it was conducted in hospitalized patients, meaning that our observations may not apply to the whole spectrum of patients who are seeking medical care and are suffering from COVID-19 pneumonia without respiratory failure. Furthermore, the time when a patient decides to seek medical help relies mainly on subjective reasons, and this may be considered as a possible bias. However, the fact that we probably investigated patients with “high risk” SARS-CoV-2 infection (for which the decision for hospitalization appears to be a realistic though doubtful choice) supports the clinical relevance of our findings. Far from being conclusive, our observations pave the way for prospective studies on patients with COVID-19 pneumonia (but without respiratory failure) seeking care in EDs or primary care facilities, which will define admission criteria for moderate COVID-19.

In conclusion, we herein described the clinical, epidemiological and laboratory characteristics of patients with moderate COVID-19 at presentation, and we demonstrated that the severity of respiratory disease (involvement of all four chest X-ray quartiles, higher respiratory rate and lower SatO_2_), older age, higher systolic blood pressure and lower blood lymphocyte count are associated with the development of respiratory failure and high risk of intubation or death in this distinct population. Although the decision for hospital admission is personalized and relies mainly on the physician’s clinical judgment, the presence of one or more of the aforementioned risk factors for the development of respiratory failure may help physicians identify patients with moderate COVID-19, who are in need of hospitalization. However, our study is “hypothesis generating” and validation of our findings with prospective studies is further required.

## Figures and Tables

**Table 1:** **Clinical and epidemiological characteristics of patients on** **admission.**

**BMI**		**Coronary Artery Disease**	41 (10.68)	**Any Cancer**	26 (6.77)
<18	1 (0.26)	**Atrial Fibrillation**	23 (6)	Active	16 (4.18)
18–24.9	191 (50.39)	**COPD**	11 (2.86)	History	10 (2.6)
25–30	118 (31.13)	**Asthma**	17 (4.43)	**Chronic Kidney Disease**	
>30	69 (18.2)	**Tuberculosis**	1 (0.26)	CKD without dialysis	20 (5.2)
**Smoking Status**		**Immuno-supression**	25 (6.51)	CKD-dialysis	9 (2.34)
Never	282 (73.44)	**Diabetes mellitus**	81 (21.09)	**Cerebrovascular disease**	18 (4.69)
Current	35 (9.11)	Type I	12 (3.13)	**Chronic liver disease**	12 (3.12)
Former	67 (17.45)	Type II	69 (17.97)	HBV	6 (1.56)
**Hypertension**	129 (33.59)	**Connective tissue disease**	17 (4.43)	HCV	4 (1.04)
				Other	2 (0.52)

Data are presented as n (%). BMI: body mass index; COPD: chronic obstructive pulmonary disease; CKD: chronic kidney disease; HBV: hepatitis virus B; HCV: hepatitis virus C.

**Table 2:** Symptoms and signs on admission.

**Sore throat/nasal congestion**	27 (7.03)	**Malaise**	114 (29.69)	**SatO_2_%**	95.7 (94–97)
**Cough**	221 (57.55)	**Headache**	35 (9.11)	**Systolic BP mmHg**	120 (110–130)
**Fever**	324 (84.37)	**Chest pain**	44 (11.46)	**Chest X-ray quartiles**	
**Diarrhea/** **Vomiting**	69 (17.97)	**Abdominal pain**	25 (6.51)	1	74 (19.27)
**Dyspnoea**	73 (19.01)	**Temperature °C**	37 (36.6–38)	2	173 (45.05)
**Confusion**	14 (3.64)	**RR (breaths/min)**	20 (18–22)	3	71 (18.49)
**Myalgia**	55 (14.32)	**BPM**	87 (78–97)	4	66 (17.19)

Quantitative variables are presented as median (inter-quartile range-IQR) and qualitative variables are presented as n (%). RR: respiratory rate; BPM: beats per minute; BP: blood pressure; SatO_2_: oxygen saturation.

**Table 3:** **Laboratory values on** **admission.**

**WBC/μL**	5670 (4525–7200)	**ESR mm/h**	35 (21–50)	**ALP IU/L**	60 (48–77)
**Neutrophils/μL**	3865 (2860–5464)	**Glucose mg/dL**	108 (97.5–130)	**γGT IU/L**	29 (17–51)
**Lymphocytes/ μL**	1210 (895–1580)	**Urea mg/dL**	30 (23.5–40)	**Billirubin mg/dL**	0.47 (0.35–0.6)
**Platelets X1000/μL (IQR)**	187 (152–241.5)	**Creatinine mg/dL**	0.9 (0.8–1.1)	**LDH IU/L**	301 (235–375)
**HgB g/dL**	14 (13–15)	**Na^+^ mmol/L**	138 (135–140)	**C-Reactive Protein mg/dL**	4.3 (1.5–8.75)
**PT sec**	12.6 (12–13.3)	**K^+^ mmol/L**	4.3 (3.9–4.7)	**CPK IU/L**	104 (68–196.5)
**aPTT sec**	31.8 (28.87–35.62)	**Corrected Ca^2+^**	8.87 (8.6–9.18)	**Cardiac troponinepg/mL**	8 (4–15)
**Fibrinogen mg/dL**	521 (446–627)	**Albumin g/dL**	4 (3.6–4.2)	**PaO_2_ mmHg**	70 (64–80)
**D-Dimers μg/mL**	0.68 (0.46–1.2)	**AST IU/L**	31 (23–45)	**P/F Ratio**	331 (301.5–381)
**Ferritin ng/mL**	270 (149–540.3)	**ALT IU/L**	24 (16.5–38)	**Lactate mmol/L**	1 (0.8–1.25)

Values are presented as median (interquartile range-IQR). WBC: white blood cells; HgB: hemoglobin; PT: prothrombin time; aPTT: activated partial thromboplastin time; ESR: erythrocyte sedimentation rate; AST: aspartate transaminase; ALT: alanine transaminase; ALP: alkaline phosphatase; γGT: gamma-glutamyl transferase; LDH: lactate dehydrogenase; CPK: creatine phosphokinase.

**Table 4:** **Major complications during** **hospitalization.**

**Hospital-Acquired Infection**	52 (13.65)	**Liver Dysfunction**		**Cardiac**	**25 (6.51)**
Pneumonia	17 (4.46)	AST/ALT elevation	52 (13.54)	pericarditis	6 (1.56)
UTI	20 (5.25)	γGT/ALP elevation	11 (2.86)	arrhythmia	11 (2.86)
Bacteremia/ Fungemia	23 (6.04)	both	36 (9.37)	ACS	3 (0.8)
C. Dif.	6 (1.57)	**Thromboembolism**		myocarditis	3 (0.8)
**Shock**	25 (6.51)	DVT	3 (0.8)	2 or more	2 (0.52)
**AKI**	23 (5.99)	PE	8 (2.08)	**Ketoacidosis**	3 (0.8)

Values are presented as n (%). UTI: urinary tract infection; C. Dif: clostridium difficile colitis; AST: aspartate transaminase; ALT: alanine transaminase; ALP: alkaline phosphatase; γGT: gamma-glutamyl transferase; ACS: acute coronary syndrome; AKI: acute kidney injury; DVT: deep venous thrombosis; PE: pulmonary embolism.

## Data Availability

Data are available upon contact with the corresponding author.

## Supplementary Materials

The following supporting information can be downloaded at: https://www.mdpi.com/article/10.3390/jpm12091472/s1, Table S1: Univariate analysis-Association with development of respiratory failure. Table S2: Univariate analysis-Association with intubation. Table S3: Univariate analysis-Association with death.
