# Supplementary material for: Moderate COVID-19: Clinical Trajectories and Predictors of Progression and Outcomes
Source: J Pers Med. 2022 Sep 8;12(9):1472. doi: 10.3390/jpm12091472 (PMC9505620; doi:10.3390/jpm12091472)
Supplement: Supplementary file 1 [file jpm-12-01472-s001.zip › jpm-1888635-supplementary.pdf]

Table S1

Dependent Variable: **Development of Respiratory Failure**					
Independent Variable	Odds ratio (95% CI)	P value	Independent Variable	Odds ratio (95% CI)	P value
**Age** (years)	1.029 (1.01-1.04)	<0.001	**Chest X-ray** (affected quartiles=3)	2.96 (1.49-5.87)	0.002
**Ethnicity** (Central Asian)	0.32 (0.16-0.61)	0.001	**Chest X-ray** (affected quartiles=4)	5.59 (2.57-12.14)	<0.001
**Ethnicity** (Middle eastern)	0.24 (0.06-0.94)	0.04			
**BMI** (>30)	1.89 (1.07-3.33)	0.028	**Cough**	1.55 (1.02-2.33)	0.038
**Arterial Hypertension**	2.019 (1.29-3.16)	0.002	**Fever**	2.7 (1.53-4.76)	0.001
**Diabetes mellitus**	2.18 (1.27-3.74)	0.005	**Confusion**	9.58 (1.24-74.01)	0.03
**CCI**	1.21 (1.09-1.34)	<0.001	**Abdominal Pain**	0.3 (0.13-0.72)	0.007
**Temperature** (^{o} C)	1.44 (1.13-1.83)	0.003	**Sore throat/ Nasal Congestion**	0.38 (0.17-0.86)	0.02
**Respiratory Rate** (breaths/min)	1.32 (1.23-1.42)	<0.001	**NEWS2** (>5)	5.54 (3.05-10.08)	<0.001
**Systolic Blood Pressure** (mmHg)	1.02 (1-1.34)	0.001	**Total Blood Lymphocytes** (cells/μl)	0.999 (0.998-1)	<0.001
**SatO_2** (%)	0.58 (0.51-0.66)	<0.001	**Albumin** (g/dL)	0.38 (0.23-0.63)	<0.001
**PaO_2** (mmHg)	0.9 (0.88-0.92)	<0.001	**AST** (IU/L)	1.01 (1-1.02)	0.01
**P/F ratio**	0.977 (0.972-0.982)	<0.001			

Table S2

Dependent Variable: **Intubation**					
Independent Variable	Odds ratio (95% CI)	P value	Independent Variable	Odds ratio (95% CI)	P value
**Age** (years)	1.04 (1.02-1.07)	0.002	**Chest X-ray** (affected quartiles=3)	9.27 (1.13-76.15)	0.038
**Coronary Artery Disease**	5.75 (2.35-14.05)	<0.001	**Chest X-ray** (affected quartiles=4)	11.53 (1.42-93.64)	0.022
**Arterial Hypertension**	2.27 (1-5.13)	0.049	**Confusion**	9.72 (2.98-31.72)	<0.001
**Diabetes mellitus**	2.7 (1.17-6.27)	0.02	**aPTT** (sec)	1.03 (1.01-1.06)	0.003
**CCI**	1.44 (1.24-1.67)	<0.001	**Urea** (mg/dL)	1.014 (1-1.02)	0.005
**Cerebrovascular disease**	6.65 (2.16-20.5)	0.001	**Creatinine** (mg/dL)	1.29 (1.08-1.54)	0.005
**Chronic Kidney Disease**	4.61 (1.68-12.67)	0.003	**Albumin (**g/dL)	0.3 (0.13-0.67)	0.003
**Atrial Fibrillation**	3.58 (1.11-11.52)	0.032			

Table S3

Dependent Variable: **Death**					
Independent Variable	Odds ratio (95% CI)	P value	Independent Variable	Odds ratio (95% CI)	P value
**Age** (years)	1.1 (1.07-1.14)	<0.001	**Confusion**	9.16 (2.97-28.26)	<0.001
**Days from symptom onset**	0.79 (0.7-0.89)	<0.001	**Lactate** (mmol/L)	3.07 (1.66-5.69)	<0.001
**Coronary Artery Disease**	5.1 (2.27-11.47)	<0.001	**Hgb** (g/dL)	0.7 (0.59-0.84)	<0.001
**Immuno-suppression**	3.73 (1.38-10.1)	0.01	**aPTT** (sec)	1.04 (1.01-1.06)	0.002
**Diabetes mellitus**	2.57 (1.22-5.38)	0.013	**D-dimers** (μg/ml)	1.21 (1.05-1.38)	0.006
**Malignancy** (active)	13.28 (4.6-38.37)	<0.001	**Urea** (mg/dL)	1.03 (1.02-1.04)	<0.001
**Cerebrovascular disease**	6.03 (2.1-17.3)	0.001	**Creatinine** (mg/dL)	1.34 (1.13-1.6)	0.001
**Chronic Kidney Disease**	7.26 (3.04-17.34)	<0.001	**Albumin** (g/dL)	0.14 (0.07-0.31)	<0.001
**Atrial Fibrillation**	7.15 (2.76-18.47)	<0.001	**Corrected** Ca^{2+}	2.71 (1.5-4.9)	0.001
**CCI**	1.92 (1.61-2.3)	<0.001	**ALT** (IU/L)	0.97 (0.95-1)	0.039

Table S1: Univariate analysis-Association with development of respiratory failure. Clinical characteristics and laboratory values obtained at presentation were independently evaluated for association with the development of respiratory failure. Calculated estimates are presented as odds ratio (OR), with 95% confidence intervals (CI). P values <0.05 were considered significant. For quantitative variables OR reflects changes per given unit. BMI: Body Mass Index, CCI: Charlson Comorbidity Index, $SatO_2$: Oxygen Saturation, PaO_2: Partial Arterial pressure of Oxygen, P/F ratio: PaO2/FiO2, NEWS2: National Early Weaning Score 2, AST: Aspartate Transaminase.

Table S2: Univariate analysis-Association with intubation. Clinical characteristics and laboratory values obtained at presentation were independently evaluated for association with intubation rate. Calculated estimates are presented as odds ratio (OR), with 95% confidence intervals (CI). P values <0.05 were considered significant. For quantitative variables OR reflects changes per given unit. CCI: Charlson Comorbidity Index, aPTT: activated partial thromboplastin time.

Table S3: Univariate analysis-Association with death. Clinical characteristics and laboratory values obtained at presentation were independently evaluated for association with mortality. Calculated estimates are presented as odds ratio (OR), with 95% confidence intervals (CI). P values <0.05 were considered significant. For quantitative variables OR reflects changes per given unit. CCI: Charlson Comorbidity Index, HgB: Hemoglobin, aPTT: activated partial thromboplastin time, ALT: Alanine Aminotransferase.
